# Facile Conversion of Polystyrene Waste into an Efficient Sorbent for Water Purification

**DOI:** 10.3390/polym14214477

**Published:** 2022-10-22

**Authors:** Cuizhu Ye, Ziyan Pan, Yi Shen

**Affiliations:** School of Food Science and Technology, South China University of Technology, Guangzhou 510640, China

**Keywords:** sulfonated polystyrene, water treatment, Pb^2+^, lysozyme and methylene blue

## Abstract

In this work, we convert a plastic waste, i.e., polystyrene (PS), into a sorbent by a simple sulfonation process. The sulfonation time was optimized and the structures of the resulting sulfonated polystyrene (SPS) was characterized by field emission scanning electron microscopy, energy-dispersive X-ray and contact angle tests. The results showed that the sulfonation time of 7 h can introduce abundant sulfonic groups and preserve the self-standing structure. Additionally, the SPS has a three-dimensional porous structure and hydrophilic surface because of the presence of numerous sulfonic groups, which could serve as effective binding sites for immobilizing varying pollutants. Furthermore, as a proof-of-concept, the adsorption performance of the SPS foams was evaluated using three pollutants, namely Pb^2+^, lysozyme and methylene blue. The adsorption isotherms were fitted by the Langmuir and Freundlich models, while the kinetics of the adsorption processes were analyzed using the pseudo-first-order, pseudo-second-order and intraparticle diffusion equations. It was found that the adsorption isotherms of Pb^2+^ and lysozyme can be better described by the Langmuir model, leading to maximum equilibrium adsorption uptakes of 10.5 and 15.7 mg g^−1^ for the adsorption of Pb^2+^ and lysozyme, respectively. Importantly, the pollutant-saturated SPS is readily regenerated by acid washing, and the recovered sorbents exhibit outstanding cyclic performance. The abundant availability of feedstock, facile preparation and regeneration processes render the SPS foams a promising sorbent for practical applications.

## 1. Introduction

With the growth of the global population and the aggravation of environmental pollution, an ever-increasing demand for clean water will be witnessed in the coming decades. Effective removal of pollutants from water is of paramount importance for water security [1,2,3]. In this context, many water treatment technologies, such as coagulation–flocculation, advanced oxidation, membrane filtration and sorption, have been extensively explored to remove pollutants from water sources [4,5,6,7]. Of these techniques, sorption is considered to be one of the most promising approaches because of its simplicity and low cost [8,9,10,11,12,13]. Since the sorbent plays a key role in the sorption process, developing high-performance sorbents is crucial for the feasibility of applying sorption in water cleanup. Thus far, numerous sorbents, such as carbons [14,15,16,17,18], oxides [19,20,21,22,23,24,25], nitrides [26,27,28,29] and polymers [30,31,32,33,34,35], have been reported. For example, Deng et al. prepared thio-functionalized polyacrylonitrile fiber for the selective and enhanced adsorption of mercury and cadmium from water [30]. Moreover, Sapurina et al. expounded the sorbents used for water purification based on conjugated polymers [34]. Unfortunately, most of these reported sorbents suffer a common drawback, i.e., high costs. In particular, the complicated fabrication process of these materials poses a great obstacle for large-scale application. As a result, high-performance and cost-effective sorbents are highly desirable, but unfortunately still lacking.

Our has group devoted many efforts to searching for sorbents for water clean-up [36,37,38,39]. We fabricated three-dimensional hierarchical architectures by integrating carbon nanofibers and graphene nanosheets into macroscopic graphite felt supports [36,37]. The resulting composite monoliths showed excellent adsorption performance for the removal of heavy metal ions, dyes and organic solvents from aqueous solutions. In addition, we prepared hierarchical magnetic carbon nanosheet assemblies, which showed remarkable adsorption uptakes of 453 and 724 mg g^−1^ for the adsorption of Pb^2+^ and Congo red, respectively [38].

Recently, we fabricated mono-dispersed sulfonated polystyrene (SPS) nanospheres for water treatment, which could effectively reduce the concentration of heavy metal ions in the solution to ppb levels in several minutes [39]. However, the widespread application of SPS nanospheres is limited because of their cost and the tedious multi-step preparation process. Moreover, SPS nanospheres are always used in powder form and must be collected from aqueous solutions by filtration after the adsorption process, which consumes additional time and manpower, further increasing the overall operating cost of the sorption process.

Polystyrene (PS), a polymer synthesized by the free radical polymerization of a styrene monomer, is extensively employed in plates, utensils (single-use), packing, toys, DVD cases and foam coffee cups [40]. However, the disposal of these products creates environmental pollution because of their nondegradable nature [41]. Additionally, the recycling of PS is currently costly and quite limited, particularly in some developing countries, which leads to serious “white pollution”. The growing scientific research elucidates the consequences of PS for wildlife animals and their habitats [42]. Given the concept of changing waste into treasure, it is of great significance to develop valuable products from PS waste [43,44,45].

Motivated by these critical issues, in this work, we utilized PS waste as a feedstock to fabricate a cost-effective sorbent via a simple sulfonation process. PS foams are widely used in the packaging industry. To this end, herein, we convert spent PS foams into a cost-effective sorbent via a simple sulfonation process. The preparation process is simple and does not involve any sophisticated instruments or complicated fabrication process. The resulting SPS sorbent exhibits a self-standing porous structure, greatly facilitating the separation and regeneration processes, which is highly favorable for practical applications.

## 2. Materials and Methods

### 2.1. Materials

Sodium hydroxide (≥96.0%) and anhydrous ethanol (≥99.7%) were purchased from the Nanjing Chemical Reagent Co. Ltd., Nanjing, China. Sulfuric acid (98.0%) and lead nitrate (≥99.0%) were purchased from the Damao Chemical Reagent Co. Ltd., Tianjin, China. Hydrochloric acid (1%) was purchased from the Guangdong Minggu Chemical Technology Co. Ltd., Shanghai, China. Lysozyme and methylene blue (MB) were purchased from the Shanghai Boao Biotechnology Co. Ltd., Shanghai, China. Spent PS foams were collected, rinsed with ethanol and dried at 60 °C in a vacuum overnight.

### 2.2. Synthesis of SPS Foams

Expanded PS foams were cut into pieces with dimensions of 5 × 5 × 2 cm and immersed in concentrated H_2_SO_4_. The sulfonation process was conducted at 60 °C with varying reaction times of 3, 4, 5, 6, 7 and 8 h. Subsequently, the resulting SPS foams were taken out from the H_2_SO_4_ solution, thoroughly washed with deionized water and then dried at 50 °C using a vacuum oven overnight. It is noteworthy that the concentrated H_2_SO_4_ can be reused in the preparation process, which greatly reduces the consumption of H_2_SO_4_.

### 2.3. Determination of Degree of Sulfonation (DoS)

The DoS of the resulting SPS foams was determined by titration. The details of the experimental procedures are presented as follows. A piece of SPS foam was immersed in 80 mL of NaOH solution for 2 days. Afterwards, 20 mL of NaOH solution was withdrawn and titrated using HCl solution. The consumed volume of HCl solution (*V*_HCl_) was recorded. The DoS was calculated by following Equation (1):

(1) $$DoS=\frac{80\times 10^{-3}\times C_{\mathrm{NaOH}}-V_{\mathrm{HCl}}\times 4\times 10^{-3}\times C_{\mathrm{HCl}}}{M}$$

where *C*_NaOH_ is the concentration of NaOH and *M* is the mass of the SPS foam.

### 2.4. Structural Characterization

The morphology of the PS and SPS foams was observed by field emission scanning electron microscopy (FESEM) (JSM-7600F, JEOL, Tokyo, Japan). The elemental composition of the samples was determined by energy-dispersive X-ray (EDX) analyses. The wetting property of the samples was analyzed by contact angle (CA) tests (JGW-360C, Chenghui Testing Machine Co. Ltd., Chengde, China). Distilled water droplets were used as a probe to study the surface properties of the foams.

### 2.5. Batch Adsorption Tests

In a typical batch adsorption test, a piece of SPS foam was immersed in a pollutant solution and then agitated at 180 rpm using a mechanical shaker at 25 °C. At given time intervals, a certain volume of aliquot was sampled and filtered through a membrane filter to remove any impurities. The concentration of Pb^2+^ was monitored using an atomic absorption spectrometer (AAS) (Z-2000 Hitachi, Hitachi, Tokyo, Japan), while those of lysozyme and MB were determined by a UV–visible spectrophotometer (North Point Rayleigh UV-1801, Beijing, China). The adsorption uptake of the adsorbent *q_t_* (mg g^−1^) at time *t* (min) was calculated by Equation (2):

(2) $$q_{t}=\frac{\left(C_{0}-C_{t}\right)\times V}{W}$$

where *C*_0_ (mg L^−1^) is the initial pollutant concentration and *C_t_* (mg L^−1^) is the concentration at time *t* (min) in the liquid phase, *V* (L) is the volume of the solution and *W* (g) is the weight of the sorbent. To determine the equilibrium adsorption capacity *q_e_* (mg g^−1^), the sorbent was immersed in the pollution solution for at least 12 h to achieve the equilibrium state of adsorption.

### 2.6. Filtration Adsorption Tests

To study the filtration performance, the SPS foams with a diameter of 1.6 cm and a thickness of 1 cm were fitted into a funnel. MB (initial concentration = 50 mg L^−1^), lysozyme (initial concentration = 150 mg L^−1^) and Pb^2+^ (initial concentration = 50 mg L^−1^) solutions were separately filtered through the foams. The concentrations of the pollutants in the effluents were analyzed.

### 2.7. Cyclic Adsorption Tests

To evaluate the cyclic adsorption performance, SPS foams were immersed in 50 mg L^−1^ pollutant (MB or Pb^2+^) solutions. The adsorption process was conducted at 25 °C for at least 12 h. Subsequently, the pollutant-saturated SPS foams were taken out from the solution and rinsed with 100 mL of 1 M HCl solution three times. The adsorption–desorption process was repeated five times. The adsorption uptakes of the SPS foams were recorded as a function of the cycle number.

## 3. Results and Discussion

### 3.1. Structural Characterization

**Figure 1:**
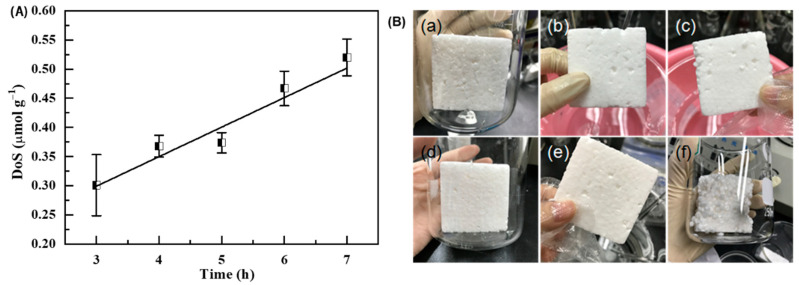
(**A**) DoS as a function of sulfonation time, and (**B**) digital photos of SPS foams with sulfonation times of (**a**) 3, (**b**) 4, (**c**) 5, (**d**) 6, (**e**) 7 and (**f**) 8 h.

Since the number of sulfonic groups in the SPS foams plays a decisive role in the adsorption performance, the sulfonation time was optimized during the preparation process. Figure 1a shows the degree of sulfonation (DoS) of the foams as a function of reaction time. The DoS values increase with increasing reaction time. Notably, the DoS of the SPS foam reached 0.52 μmol g^−1^ when the sulfonation process was conducted for 7 h. Figure 1b shows the corresponding digital photos of the foams. It reveals that the resulting SPS foams have an intact self-standing structure when the sulfonation time is less than 8 h. When the sulfonation time is over 8 h, the SPS foams start to collapse, leading to many isolated particles with sizes of several millimeters. Although these particles possess large DoS values, they are quite difficult to separate from the solutions, which greatly limits their practical applications. In contrast, the bulky free-standing SPS foams afford a significant advantage during separation. Under this circumstance, the sulfonation time is optimized to be 7 h in this work.

**Figure 2:**
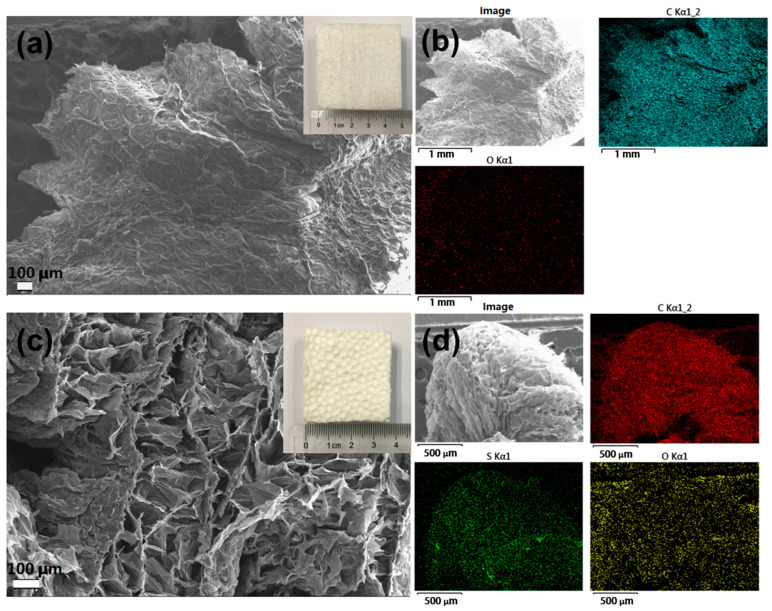
FESEM images of (**a**) pristine EPS and (**c**) SPS foams (the insets in (**a**,**c**) are the corresponding digital photos of EPS and SPS foams); EDX mapping images of (**b**) pristine EPS and (**d**) SPS foams.

The morphology of the foams was observed by FESEM, as shown in Figure 2. The pristine PS foam possesses a macroporous structure, as shown in Figure 2a. The EDX results indicate that the pristine PS foam contains carbon and oxygen elements, as shown in Figure 2b. Figure 2c shows the morphology of the resulting SPS foams. It clearly reveals that the porous structure is well preserved in the SPS foams. Apart from carbon and oxygen, sulfur is also detected by the EDX analyses, as shown in Figure 2d. The insets shown in Figure 2a,c are the digital photos of the PS and SPS foams, respectively. The pristine PS foam has dimensions of ca. 5 × 5 × 2 cm. After the sulfonation and drying processes, the contraction of the foam is noted, resulting in a smaller size of the SPS foam. A close inspection could reveal that the surface of the SPS foams is much rougher than that of the pristine PS foams. This could also be related to the sulfonation process.

**Figure 3:**
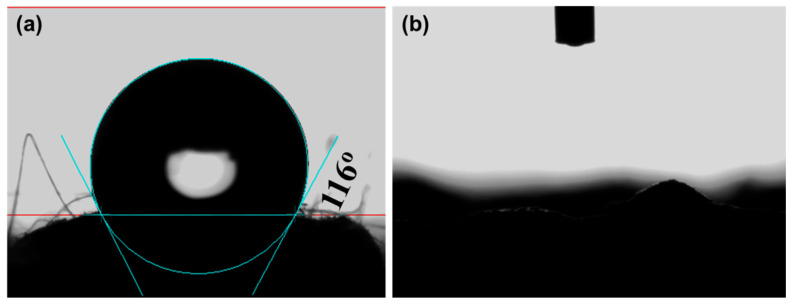
CA tests of (**a**) pristine PS and (**b**) SPS foams.

The wettability of the foams was investigated by the CA tests, as shown in Figure 3. The CA of the pristine PS foam is determined to be 116° (see Figure 3a), indicating the hydrophobic properties of the PS foam. In contrast, the water droplet readily penetrates the SPS foam (see Figure 3b and the Video S1 in the Supporting Information), reflecting the hydrophilic surface of the SPS foam. The variations in surface properties are attributed to the introduction of hydrophilic sulfonic groups and the increased surface roughness arising from the sulfonation process [46].

### 3.2. Adsorption Performance

The adsorption performance of the resulting SPS foams was evaluated using three typical substrates, including a heavy metal pollutant (i.e., Pb^2+^), a protein (i.e., lysozyme) and a dye (i.e., MB). The adsorption characteristics of the SPS foams toward these three substrates were extensively investigated. A piece of SPS foam was immersed in the pollutant solution with different initial concentrations. After reaching adsorption equilibrium, the concentration of Pb^2+^ was analyzed by an inductive plasma emission spectroscope, while those of lysozyme and MB were determined by a UV–visible spectrophotometer. Figure S1 (from the Supplementary Materials) shows the UV spectra of MB. It is noteworthy that several peaks are seen in the UV spectra of MB. The strongest peak, located at ca. 664 nm, was employed to determine the concentration of MB. A broad peak located at ca. 280 nm was observed from the UV spectra of lysozyme, as shown in Figure S2. In the testing concentration range, the peak absorbance is well correlated with the concentration, as shown in Figure S1b and S2b.

**Figure 4:**
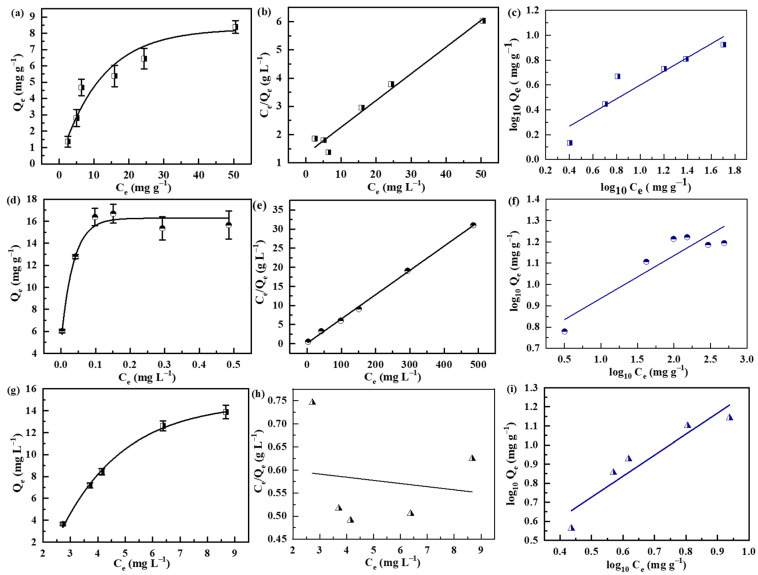
(**a**,**d**,**g**) Adsorption isotherms, (**b**,**e**,**h**) Langmuir fitting curves and (**c**,**f**,**i**) Freundlich fitting curves of the adsorption of pollutants by SPS foams. Square: Pb^2+^, circle: lysozyme and triangle: MB.

The adsorption isotherms were first recorded by plotting the equilibrium adsorption uptake (*q_e_* (mg g^−1^)) against the equilibrium substrate concentration (*C_e_* (mg L^−1^)). Figure 4a,d,g display the adsorption isotherms of Pb^2+^, lysozyme and MB, respectively. To determine the adsorption constant and maximum adsorption capacity, the isotherms were further analyzed by the Langmuir and Freundlich models, as expressed by Equations (3) and (4), respectively.

(3) $$q_{e}=\frac{q_{max}bC_{e}}{1+bC_{e}}$$

(4) $$q_{e}=kC_{e}^{1/n}$$

where *q_max_* (mg g^−1^) is the maximum adsorption capacity, *b* (L mg^−1^) is the Langmuir adsorption constant, and *k* (mg^1 − 1/n^·L^1/n^·g^−1^) and *n* are the Freundlich constants associated with adsorption capacity and adsorption intensity, respectively.

The resulting curves fitted by the Langmuir model are displayed in Figure 4b,e,h, while those obtained by the Freundlich model are displayed in Figure 4c,f,i. The corresponding fitting parameters and coefficients of determination (R^2^) are listed in Table 1. Modeling results reveal that the adsorption isotherms of Pb^2+^ and lysozyme can be better described by the Langmuir model, while that of MB is better described by the Freundlich model. The SPS foams possess theoretical maximum adsorption capacities of 10.5 and 15.7 mg g^−1^ for the adsorption of Pb^2+^ and lysozyme, respectively. Since the adsorption of MB cannot be fitted by the Langmuir model, its theoretical maximum adsorption capacity cannot be determined. Notably, the three pollutants used in this work are positively charged at the experimental conditions, while the surface of SPS is negatively charged owing to the sulfonic groups. Under this circumstance, the binding of the pollutants to the SPS foams is mainly attributed to the electrostatic interaction. However, since the DoS value of the SPS foam is low in this study, the π–π stacking interaction could also possibly occur in immobilizing MB to SPS foams. This could be the reason for the different adsorption isotherm of MB as compared with those of Pb^2+^ and lysozyme. It should be pointed out that although the resulting maximum adsorption capacities of the SPS foams are smaller than those of PS-based sorbents reported in the literature [47,48], the prominent advantage of the SPS foams lies in the bulky self-standing morphology, which is highly beneficial for separation but compromises their adsorption capacities.

**Figure 5:**
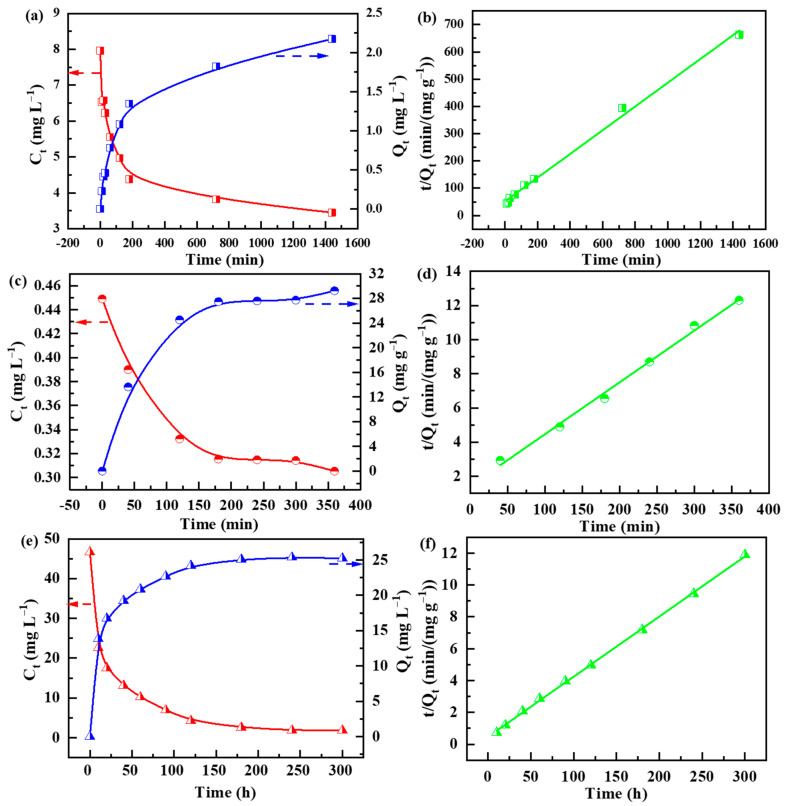
Adsorption kinetics of (**a**) Pb^2+^, (**c**) lysozyme and (**e**) MB by SPS foams, and (**b**,**d**,**f**) corresponding fitting curves obtained by the pseudo-second-order equation. (The red arrow points to the left axis, the blue arrow points to the right axis.)

The adsorption kinetics of the SPS foams were also studied. The instant concentrations and adsorption uptakes of the pollutants were recorded as a function of contact time, as shown in Figure 5. The concentrations of the pollutants (the red lines) rapidly decrease while the adsorption uptakes (the blue lines) increase during the initial period of contact time and then reach an equilibrium state. To study the kinetics of the adsorption of metal ions, three kinetic models, including the pseudo-first-order, pseudo-second-order and intraparticle diffusion models, were applied to fit the experimental data. The pseudo-first-order kinetic model can be expressed by Equation (5).

(5) $$\log\left(q_{e}-q_{t}\right)=\log q_{e}-\frac{k_{1}}{2.303}t$$

where *k_1_* (min^−1^) is the adsorption rate constant of the first-order kinetic model. The pseudo-second-order kinetic model is expressed by Equation (6).

(6) $$\frac{t}{q_{t}}=\frac{1}{k_{2}q_{e}^{2}}+\frac{1}{q_{e}}t$$

where *k*_2_ (g (mg·min)^−1^) is the adsorption rate constant of the second order kinetic model. The intraparticle diffusion model is expressed by Equation (7).

(7) $$q_{t}=k_{p}t^{1/2}+C$$

where *k_p_* (mg (g h^1/2^)^−1^) is the intraparticle diffusion rate constant and *C* (mg g^−1^) is a constant related to the thickness of the boundary layer.

The fitting curves obtained by the pseudo-first-order equation are shown in Figure 5b,d,f, while those by the pseudo-second-order and intraparticle diffusion equations are displayed in Figures S3 and S4, respectively. The resulting fitting parameters are summarized in Table 2. It reveals that among the three equations, the pseudo-second-order best describes the kinetic data. Based on the pseudo-second-order equation, the equilibrium adsorption uptakes are 33, 26.4 and 2.4 mg g^−1^, while the adsorption constants are 0.021, 0.078 and 0.0043 g (mg·min)^−1^ for the lysozyme, MB and Pb^2+^, respectively. The equilibrium adsorption uptake could be related to the molar mass and charge of the pollutants. Pb^2+^ ions have a smaller molar mass than those of lysozyme and MB. In addition, a Pb^2+^ ion has two positive charges. In contrast, only one positive charge could be found for the lysozyme and MB molecules. Thus, the binding of Pb^2+^ ions to the SPS foam could involve more SO_3_^−^ groups as compared with those of lysozyme and MB. These two aspects could result in the lowest equilibrium adsorption uptake of Pb^2+^.

**Figure 6:**
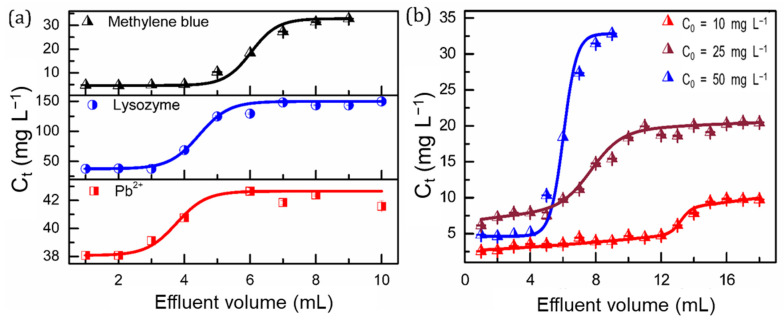
(**a**) Breakthrough curves for the penetration of the pollutants through the SPS foams and (**b**) dependence of breakthrough curves on the initial concentration of MB.

One prominent feature of the resulting SPS foam lies in its three-dimensional self-standing structure, which affords a significant advantage for fix-bed filtration adsorption. The filtration adsorption performance of the SPS foams was evaluated. Figure 6a shows the breakthrough curves for the filtration of the pollutants. The concentrations of the pollutants in the effluents present a clear step profile, indicating that the major pollutant molecules were immobilized in the sorbents in the initial filtration stage, but directly penetrated through the foams when they were saturated by the pollutants. At the breakthrough point, 5.2 mL of MB, 3.7 mL of lysozyme and 3.0 mL of Pb^2+^ solution were filtrated through the foams. Figure 6b shows the concentration profiles of MB in the effluents with different initial pollutant concentrations. With an increasing initial concentration, the volume of the effluent decreases at the breakthrough point. For instance, at the breakthrough points, the effluent volumes are determined to be 5.2, 6.3 and 12.5 mL at initial concentrations of 50, 25 and 10 mg L^−1^, respectively.

**Figure 7:**
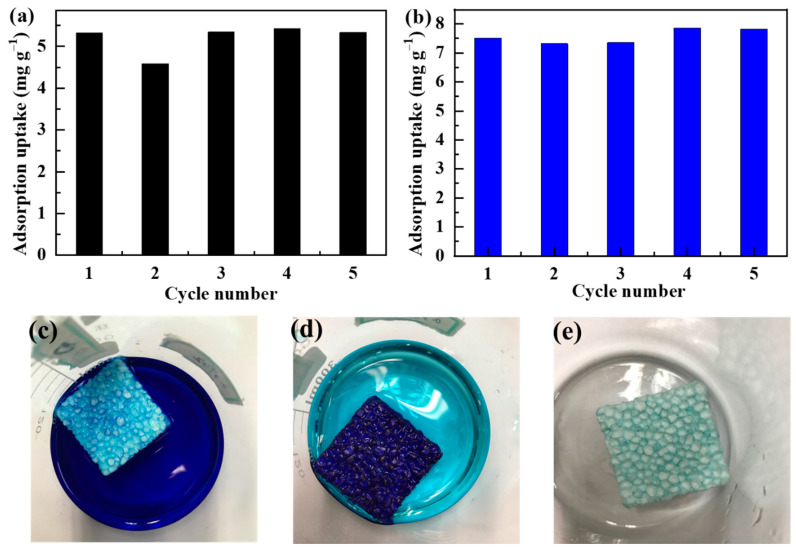
Cyclic adsorption of (**a**) Pb^2+^ and (**b**) MB, and digital photos of SPS (**c**) before and (**d**) after adsorption of MB, and (**e**) after desorption.

For practical applications, the cyclic performance of the sorbents is also critical. To this end, the desorption of the Pb^2+^- and MB-saturated SPS foams was conducted by immersing them in 1 M HCl. The regenerated SPS forms were re-utilized to remove the pollutants from aqueous solutions. This adsorption–desorption process was repeated five times and the adsorption uptakes were recorded. Figure 7a,b show the adsorption uptakes of Pb^2+^ and MB, respectively, as a function of cyclic number. It reveals that the adsorption uptakes are quite stable, indicating the outstanding cyclic performance of the SPS foams. Figure 6c–e show the digital photos of SPS before and after the adsorption of MB and after desorption, respectively, which vividly reveal the adsorption and desorption processes. It is noteworthy that the regeneration process is quite easy and does not involve any separation process because of the bulky three-dimensional structure of the SPS foams.

## 4. Conclusions

In summary, we employed PS waste as a feedstock and converted it into bulky SPS sorbents via a facile sulfonation process. Using Pb^2+^, lysozyme and MB as representative substrates, the characteristics of the adsorption processes were studied. The adsorption isotherms of Pb^2+^ and lysozyme can be better described by the Langmuir model, leading to maximum equilibrium adsorption uptakes of 10.5 and 15.7 mg g^−1^ for the adsorption of Pb^2+^ and lysozyme, respectively. In contrast, the isotherm of MB is better described by the Freundlich model. For all three substrates, the kinetics of the adsorption processes were best described by the pseudo-second-order equation. The electrostatic interaction was identified as the main mechanism of the adsorption process. The Pb^2+^- and MB-saturated SPS foams can be readily regenerated by immersing them in HCl solution and the recovered sorbents show excellent cyclic adsorption performance. The most prominent features of the SPS sorbent reported in this work lie in the bulky three-dimensional structure, which avoids the tedious separation process. Additionally, the simple preparation process and abundant availability of PS waste as a feedstock indicate SPS foams to be a promising sorbent for practical applications.

## Figures and Tables

**Table 1:** Fitting the equilibrium data using the Langmuir and Freundlich models.

Sorbates	Langmuir Model			Freundlich Model	
	R^2^	q_max_	b	R^2^	k
Lysozyme	0.9981	15.7	0.48	0.8079	5.42
MB	−0.3031	−146.8	−0.011	0.8608	1.50
Pb^2+^	0.9633	10.5	0.071	0.9311	1.11

**Table 2:** Kinetic fitting results of the adsorption processes.

Sorbates	Pseudo-First-Order			Pseudo-Second-Order			Intraparticle Diffusion	
	R^2^	q_e_	k_1_	R^2^	q_e_	k_2_	R^2^	k
Lysozyme	0.8747	21.29	0.01	0.9937	33.0	0.021	0.9003	1.55
MB	0.9745	18.42	0.025	0.9992	26.4	0.078	0.7243	1.22
Pb^2+^	0.8967	1.74	0.002	0.9930	2.4	0.0043	0.9126	0.057

## Data Availability

Not applicable.

## Supplementary Materials

The following supporting information can be downloaded at: https://www.mdpi.com/article/10.3390/polym14214477/s1, Video S1 showing the penetration of water droplets into SPS foams. UV spectra of methylene blue and lysozyme and resulting calibrated curves are shown in Figures S1 and S2. Adsorption fitting curves of Pb^2+^, lysozyme and methylene blue by the pseudo-first-order equation and intraparticle diffusion equation are shown in Figures S3 and S4.
