# Supplementary material for: Facile Conversion of Polystyrene Waste into an Efficient Sorbent for Water Purification
Source: Polymers (Basel). 2022 Oct 22;14(21):4477. doi: 10.3390/polym14214477 (PMC9655115; doi:10.3390/polym14214477)
Supplement: Supplementary file 1 [file polymers-14-04477-s001.zip › Supporting Information.pdf]

Supporting Information

Facile Conversion of Polystyrene Waste into an Efficient Sorbent for Water Purification

Cuizhu Ye,[†, 1] Ziyan Pan,[†, 1] and Yi Shen[*, †]

[†] School of Food Science and Technology, South China University of Technology, Guangzhou, 510640, China.

[1] The authors contributed equally to this work.

Correspondence: *feyshen@scut.edu.cn (Y. S.)

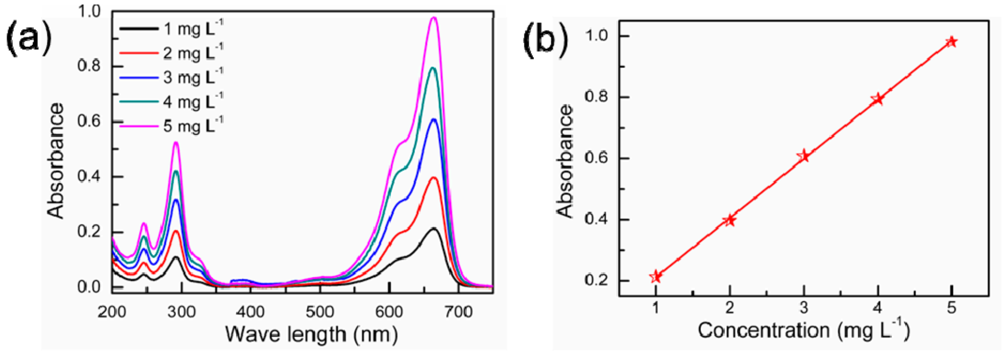

Figure S1 (a) UV spectra of methylene blue, and (b) resulting calibrated curves.

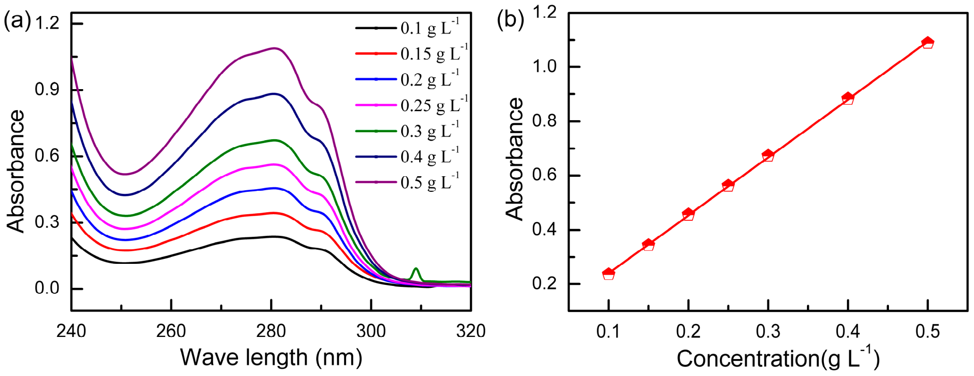

Figure S2 (a) UV spectra of lysozyme, and (b) resulting calibrated curves.

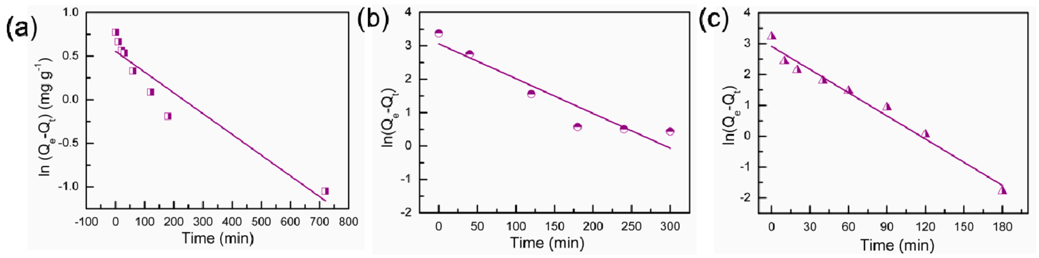

Figure S3 Adsorption fitting curves of (a) Pb^{2+}, (b) lysozyme and (c) methylene blue by the pseudo-first-order equation.

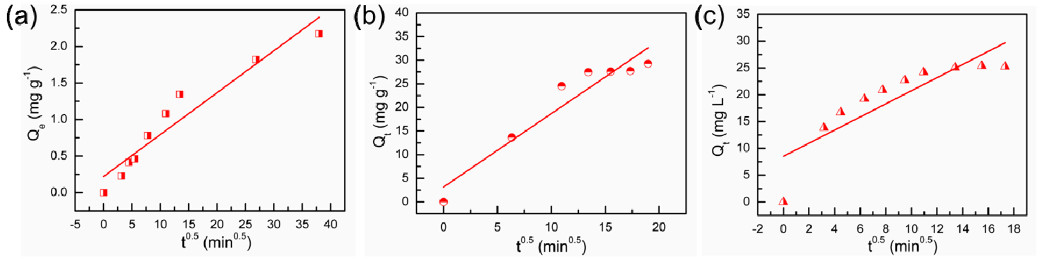

Figure S4 Adsorption fitting curves of (a) Pb^{2+}, (b) lysozyme and (c) methylene blue by intraparticle diffusion equation.
